# Pessaries

**Published:** 1878-11

**Authors:** 


Pessaries.—In regard to the use of pessaries: If, on making conjoined manipulation, or passing the uterine probe, the fundus does not remain in position after an instrument of this kind has been introduced, take it out at once; and if you cannot get the fundus into its proper place, then do not put in any pessary at all. At the end of forty-eight hours, or at all events, within a very few days after the introduction of the pessary, you should always make an examination, and if you find that it is not holding the uterus up in good position; you should remove it and put in another. If you cannot find one that will do this properly, it is a great deal better that the patient should be left without any instrument whatever in her vagina.—*Med. and Surg. Reporter.*

